# Evaluation of growth, physiological response, and drought resistance of different flue-cured tobacco varieties under drought stress

**DOI:** 10.3389/fpls.2024.1442618

**Published:** 2024-09-26

**Authors:** Yi-nan Zhang, Ye Zhuang, Xiao-guo Wang, Xiao-dong Wang

**Affiliations:** ^1^ Henan Province Dryland Agricultural Engineering Technology Research Center/College of Agronomy, Henan University of Science and Technology, Luoyang, Henan, China; ^2^ Technology Research Center, Henan Tobacco Company, Luoyang, Henan, China

**Keywords:** tobacco varieties, drought tolerance, antioxidant enzymes, principal component analysis, comprehensive evaluation

## Abstract

**Background:**

In recent years, more severe droughts have occurred frequently in many parts of the world, drought stress is the primary abiotic stress factor restricting the growth and quality of flue-cured tobacco. Therefore, screening dryland cultivation-compatible flue-cured tobacco varieties will help reduce the negative impact of drought.

**Methods:**

Tobacco varieties were selected: Qinyan 96 (Q96), Zhongyan 101 (Z101), Yunyan 87 (Y87), and Yunyan 116 (Y116). A pot experiment was conducted with four water supply gradients: sufficient, mild stress, moderate stress, and severe stress. The aim was to analyze inter-varietal differences in agronomic traits, photosynthetic traits, reactive oxygen species (ROS) metabolism, and antioxidant enzyme system under drought stress. Additionally, the drought resistance of four flue-cured tobacco varieties was evaluated using principal component analysis and membership function analysis.

**Results:**

The results showed that drought intensification inhibited seedling growth and development across all varieties, with Q96 showing the least decrease and Y116 the greatest. With the increasing degree of drought stress, photosynthetic rates (P_n_), transpiration rate (T_r_), and stomatal conduction (G_s_) have shown gradually decreasing trends, while substomatal cavity CO_2_ concentration (C_i_) showed a growing trend. Severe drought corresponded with lower chlorophyll content and decreased the maximal photochemical efficiency (F_v_/F_m_), photosystem II (PSII), and photochemical quenching coefficient (qP) in all varieties, while steady-state non-photochemical quenching (NPQ) increased. Increased drought stress led to significantly higher reactive oxygen species (ROS) and malondialdehyde (MDA) content accumulation in tobacco seedlings. The antioxidant enzyme activities in, Q96, Z101, and Y87 increased under mild drought stress, whereas Y116 showed decreased activity.

**Conclusion:**

The drought resistance ranking among the four varieties is as follows: Q96 > Z101 > Y87 > Y116. Therefore, Q96 is a promising drought-tolerant breeding material that can be used as a reference for dryland cultivation of flue-cured tobacco.

## 1 Introduction

Global climate change has increased extreme weather events (Gu et al., 2020; Ji et al., 2023), with drought becoming a significant factor affecting flue-cured tobacco production (Tang et al., 2020). Drought stress can disrupt cellular metabolism, hinder growth and development, and even cause stagnation in tobacco (Petrov et al., 2015). In China, tobacco, an important economic crop, is often grown in regions prone to soil drought and water scarcity. However, tobacco requires substantial water for optimal growth and development (Biglouei et al (Biglouei et al., 2010; Xu et al., 2020). Therefore, sufficient water conditions are essential for ensuring stable yield and quality.

Drought stress significantly impacts tobacco in multiple ways. The growth and development of leaves are hindered, resulting in stunted plants with wrinkled leaves (Yang et al., 2017; Liu et al., 2021). This stress also leads to insufficient dry matter accumulation, preventing normal maturation (Su et al., 2017). Severe drought stress can cause permanent wilting and, ultimately, tobacco plant death (Negin et al., 2023). Furthermore, drought stress affects the physiological characteristics and metabolic levels of tobacco (Hu et al., 2023). During the process of plant growth and development, drought stress significantly inhibits photosynthesis (Mukarram et al., 2021). Specifically, it reduces the net photosynthetic rate, transpiration rate, and stomatal conductance, thereby weakening photosynthetic performance (Khalvandi et al., 2021). Upon exceeding the plant’s regulatory capacity under drought stress, disruption of the reactive oxygen species (ROS) system metabolism occurs, generating substantial ROS amounts (Laxa et al., 2019; Mittler et al., 2022). This results in membrane lipid peroxidation and subsequent disruption of normal cell membrane function (Mansoor et al., 2022). Drought stress of varying degrees escalates malondialdehyde (MDA) content, a membrane lipid peroxide in plants, with MDA production serving as a stress damage indicator (AlKahtani et al., 2021). Tobacco employs antioxidant enzymes for self-protection against drought stress, mitigating resultant damage (Begum et al., 2020; Rajput et al., 2021). In summary, drought-resistant tobacco varieties should exhibit strong traits such as high photosynthetic capacity and robust antioxidant enzyme activity (Xu et al., 2022).

In typical arid and semi-arid regions, water scarcity and inadequate irrigation in tobacco fields negatively impact the yield and quality of flue-cured tobacco, which hinders cultivation and reduces farmers’ income. Therefore, identifying drought-resistant flue-cured tobacco varieties and screening germplasm for breeding new cold-tolerant varieties is crucial for improving production. Recent studies have shifted from evaluating plant drought resistance based on a single growth index or physiological trait to more comprehensive analyses. For example, Wu and Bao (2012) identified 10 drought resistance indexes in winter wheat, including P_n_, POD, and MDA. Wang et al. (2022b) found that amino acid and lipid metabolism play key roles in the drought resistance of two soybean varieties. Additionally, Quevedo et al. (2022) developed a model to predict water deficit tolerance in cotton varieties. However, comprehensive evaluations of drought resistance in flue-cured tobacco varieties are limited. In our pot experiment, we used four flue-cured tobacco varieties to compare biomass accumulation, photosynthetic parameters, chlorophyll fluorescence, reactive oxygen species metabolism, and antioxidant systems under varying levels of drought stress. We then used principal component analysis and membership function to assess the drought resistance of these seedlings and identify the varieties best suited for dryland planting. Our aim is to further elucidate the drought resistance mechanisms in flue-cured tobacco seedlings and provide a foundation for breeding drought-resistant varieties.

## 2 Materials and methods

### 2.1 Varieties and experimental design

Test tobacco varieties: ‘Qinyan 96’ (Q96), ‘Zhongyan 101’ (Z101), ‘Yunyan 87’ (Y87), and ‘Yunyan 116’ (Y116).

The experiment was conducted at the Henan University of Science and Technology Experimental Farm’s dry greenhouse from early May to late June 2018. The greenhouse had a roof covered with transparent sunlight panels (> 90% light transmittance) and rolling shutters on both sides for ventilation and heat dissipation. Six thermometers were installed inside for real-time temperature monitoring. The experimental plastic basins had an upper diameter of 40 cm, a lower diameter of 25 cm, and a height of 35 cm. Each basin contained 20 kg of soil sieved through a 0.50 cm × 1 cm mesh, air-dried, and mixed with fertilizer (3.50 g of pure N per pot, N: P_2_O_5_: K_2_O = 1:1.5:1). Test fertilizers included nitrate phosphate fertilizer (32% total nitrogen, 4% available phosphorus), ammonium phosphate (11% total nitrogen, 44% available phosphorus), potassium sulfate (50%), and cake fertilizer (5% total nitrogen).

In this experiment, four treatments were established: (1) CK: sufficient water supply (70–75% soil moisture content); (2) D1: Mild stress induced (55–60% soil moisture content); (3) D2: Moderate stress (45–50% soil moisture content); and (4) D3: Severe stress (35–40% soil moisture content). Each treatment comprised 10 replicates, and one tobacco seedling was transplanted per pot, totaling 120 pots. A negative pressure gauge was installed in each pot, and tobacco seedlings of similar vigor, shape, and size were selected for transplantation (one tobacco seedling per pot). Upon completion of the seedling return period (five days post-transplantation), water control was commenced. Following seven days of drought stress treatment, agronomic traits were assessed. For physiological indicator measurements, the third fully unfolded leaf from the top was selected.

Verification of water control reliability: Before tobacco seedling transplantation, a preliminary water control treatment experiment was conducted. Each pot, pre-filled with dry soil, was equipped with a negative pressure gauge and subjected to different water gradients. Upon stabilization of the negative pressure gauge readings, the maximum field water holding capacity of the soil was determined using the ring knife method. Subsequently, the soil water potential (negative pressure gauge readings) and corresponding soil relative water content were measured for each pot. Analysis revealed a significant negative correlation between soil moisture content and soil water potential (*r* ^2^ = -0.92^**^), indicating that negative pressure meter readings effectively represent the relative soil moisture content.

### 2.2 Measurement items and methods

#### 2.2.1 Determination of growth indicators

Three representative tobacco plants of comparable growth from each treatment were selected for the examination of their agronomic traits. Simultaneously, three complete fresh tobacco plant samples (including roots, stems, and leaves) with similar growth conditions were collected from each treatment. The drying process was initiated at 105°C for 30 minutes, followed by 80°C until a constant weight was achieved. Subsequently, the dry matter accumulation of the entire tobacco plant (i.e., dry weight in grams) was weighed and calculated.

#### 2.2.2 Chlorophyll content determination

Three tobacco seedlings of similar growth from each treatment were selected. Using a leaf punch, their functional leaves (≥ 5 cm, third leaf position below the heart leaf) were sampled and measured for chlorophyll content. The chlorophyll content was determined spectrophotometrically (Vernon, 1960).

#### 2.2.3 Determination of photosynthetic parameters

The photosynthetic parameters, including photosynthetic rates (P_n_), transpiration rate (T_r_), stomatal conductance (G_s_), and substomatal cavity CO2 concentration (C_i_), were measured for the same functional leaves from the tobacco plants studied for agronomic traits using a Li-6400 XT portable photosynthetic instrument between 9:00 a.m. and 11:00 a.m. on a sunny day.

#### 2.2.4 Determination of chlorophyll fluorescence parameters

The chlorophyll fluorescence parameters, including maximal photochemical efficiency (F_v_/F_m_), photosystem II (PSII), photochemical quenching coefficient (qP), and non-photochemical quenching (NPQ), were assessed using a PAM-2100 portable modulation fluorescence meter (Walz, Germany) at the same time as the photosynthetic measurements. The parameters were calculated using the following formulas:


(1) $$F_{v}/F_{m}=(F_{m}-F_{0})/F_{m}$$

(2) PSII=(Fm'−Fs)/Fm'

(3) qP=(Fm'−Fs)/(Fm'−F0')

(4) NPQ=(Fm−Fm')/Fm'

#### 2.2.5 Determination of reactive oxygen species metabolism and antioxidant enzyme activity

Before killing the green leaves, functional leaves were selected for sampling to determine malondialdehyde concentration and antioxidant enzyme activity. Specifically, superoxide dismutase (SOD) detection utilized the nitrogen blue tetrazole photochemical reduction method, peroxidase (POD) detection employed the guaiacol method, CAT detection used the ultraviolet absorption method, and malondialdehyde (MDA) content detection relied on the thiobarbituric acid colorimetric method (Schmedes and Hølmer, 1989). The calculation of O_2_^-^ production rate was performed as per a previous method (Ai-Guo and Guang-Hua, 1990).

### 2.3 Comprehensive evaluation of drought resistance

For varieties, the membership function value for each comprehensive index should be determined. The variance contribution rate of each index should then be used to calculate the principal component weight. Finally, the comprehensive evaluation value (D value) of drought resistance should be calculated using the membership function value and principal component weight.:


(5) $$U(Xj)=(Xj-X_{min})/(X_{max}-X_{min})$$

(6) Wj=Pj/ΣPj,j=1, 2, 3,…, n

(7) D=Σ[U(X j)×Wj],j=1, 2, 3,…, n

where: Xj is the j^th^ composite index value of each variety, X_max_ and X_min_ are the maximum and minimum values of j^th^ composite index value, respectively. Wj denotes the weight of the j^th^ principal component in all principal components, and Pj is the variance contribution rate of the j^th^ principal component. U(Xj) is the value of the j^th^ principal component score functionalized by membership, and the comprehensive evaluation D value of drought resistance of the variety (line) is calculated according to equation (7). The D value of each variety (line) was ranked, and the D value range was 0.00–1.00. The higher the score, the higher the ranking, the stronger the drought resistance, and the weaker the drought resistance (Bao et al., 2023).

### 2.4 Data processing

Data processing and analysis were conducted using Excel and SPSS statistical software, while mapping was performed using Origin 2021 software. The general linear model in SPSS 23 was used to conduct one-way analysis of variance and LSD tests followed by Dunnett test at the 0.05 probability level.

## 3 Results

### 3.1 Effects of drought stress on the growth of tobacco seedlings

**Table 1** shows that as drought stress increases, the plant height, maximum leaf length, maximum leaf width, maximum leaf area, and dry weight per plant in the four flue-cured tobacco varieties progressively decrease. However, different varieties exhibit varying responses to drought stress. Under D1 treatment, compared to CK treatment, the plant height of Q96 did not significantly decrease, while Z101, Y87, and Y116 showed reductions of 9.19%, 12.76%, 13.66%, and 15.04%, respectively. Under D2 treatment, plant heights of Q96, Z101, Y87, and Y116 decreased by 20.00%, 22.66%, 26.34%, and 30.07%, respectively. Under D3 treatment, the decreases were 28.38%, 31.77%, 35.61%, and 39.86%, respectively. Similarly, under D1 treatment, the maximum leaf length of Q96 decreased significantly, while Z101, Y87, and Y116 showed reductions of 6.48%, 11.40%, 13.85%, and 17.46%, respectively, compared to the control. Under D2 treatment, the maximum leaf lengths of Q96, Z101, Y87, and Y116 decreased by 15.12%, 21.05%, 23.08%, and 25.62%, respectively. Under D3 treatment, these decreases were 22.25%, 27.85%, 33.19%, and 37.41%, respectively. Additionally, under D1 treatment, the maximum leaf width of Q96 decreased significantly, while Z101, Y87, and Y116 decreased by 5.50%, 11.21%, 13.66%, and 17.84%, respectively. Under D2 treatment, maximum leaf widths decreased by 16.97%, 21.98%, 22.91%, and 25.31%, respectively. Under D3 treatment, the decreases were 22.48%, 29.74%, 33.04%, and 36.10%, respectively.

**Table 1:** Effects of different degrees of drought stress on agronomic characters and dry weight per plant of flue-cured tobacco seedlings.

Indexes	Treatment	Variety			
		Q96	Z101	Y87	Y116
Plant height	CK	12.33 ± 0.67a	12.80 ± 0.46a	13.67 ± 0.47a	13.97 ± 0.47a
	D1	11.20 ± 0.66a	11.17 ± 0.35b	11.80 ± 0.36b	11.87 ± 0.49b
	D2	9.87 ± 0.35b	9.90 ± 0.30c	10.07 ± 0.38c	9.77 ± 0.31c
	D3	8.83 ± 0.25c	8.73 ± 0.21d	8.80 ± 0.40d	8.40 ± 0.46d
Maximum leaf length	CK	15.43 ± 0.59a	15.20 ± 0.56a	15.17 ± 0.42a	14.70 ± 0.46a
	D1	14.43 ± 0.60a	13.47 ± 0.65b	13.07 ± 0.55b	12.13 ± 0.45b
	D2	13.10 ± 0.46b	12.00 ± 0.27c	11.67 ± 0.32c	10.93 ± 0.35c
	D3	12.00 ± 0.27c	10.97 ± 0.25d	10.13 ± 0.40d	9.20 ± 0.40d
Maximum leaf width	CK	7.27 ± 0.35a	7.73 ± 0.25a	7.57 ± 0.15a	8.03 ± 0.32a
	D1	6.87 ± 0.46a	6.87 ± 0.35b	6.53 ± 0.31b	6.60 ± 0.27b
	D2	6.03 ± 0.15b	6.03 ± 0.25c	5.83 ± 0.29c	6.00 ± 0.20c
	D3	5.63 ± 0.21c	5.43 ± 0.15d	5.07 ± 0.21d	5.13 ± 0.32d
Maximum leaf area	CK	71.22 ± 5.75a	74.54 ± 1.98a	72.82 ± 2.57a	74.88 ± 1.55a
	D1	62.98 ± 6.62a	58.58 ± 0.48b	54.24 ± 4.78b	50.80 ± 2.38b
	D2	50.15 ± 2.01b	45.94 ± 2.21c	43.16 ± 1.70c	41.64 ± 2.28c
	D3	42.90 ± 2.26c	37.80 ± 1.22d	32.61 ± 2.59d	29.92 ± 0.76d
Dry weight per plant	CK	20.63 ± 0.90a	23.13 ± 1.22a	22.98 ± 0.51a	23.53 ± 0.61a
	D1	17.57 ± 0.87b	18.70 ± 0.66b	18.43 ± 0.40b	17.43 ± 0.80b
	D2	15.17 ± 0.65c	16.03 ± 0.45c	15.77 ± 0.87c	15.60 ± 0.40c
	D3	12.60 ± 0.82d	13.53 ± 1.00d	12.30 ± 0.62d	11.30 ± 0.62d

Different lowercase letters in the same column indicate significant differences among different treatments of the same variety (P< 0.05).

Compared to the CK treatment, the maximum leaf area of Q96 significantly decreased under D1 treatment, while the maximum leaf area of Z101, Y87, and Y116 decreased by 11.57%, 21.42%, 25.52%, and 32.16%, respectively. Under D2 treatment, the maximum leaf area of Q96, Z101, Y87, and Y116 decreased by 29.59%, 38.37%, 40.73%, and 44.39%, respectively. With D3 treatment, the maximum leaf area further decreased by 39.76%, 49.28%, 55.22%, and 60.05% for Q96, Z101, Y87, and Y116, respectively. Additionally, compared to CK treatment, the dry weight per plant of Q96, Z101, Y87, and Y116 significantly decreased under D1 treatment, by 14.86%, 19.16%, 19.74%, and 25.92%, respectively. Under D2 treatment, the dry weight per plant of Q96, Z101, Y87, and Y116 decreased by 26.49%, 30.69%, 31.35%, and 33.71%, respectively. Under D3 treatment, these decreases were 38.93%, 41.50%, 46.44%, and 51.98%, respectively. The results indicate that as drought stress intensifies, the reductions in plant height, maximum leaf length, maximum leaf width, maximum leaf area, and dry weight per plant worsen, ranking the drought resistance of the four flue-cured tobacco varieties as Q96 > Z101 > Y87 > Y116.

### 3.2 Effects of drought stress on chlorophyll content in tobacco seedlings

**Table 2** shows that the contents of chlorophyll a, chlorophyll b, and chlorophyll a+b in the seedlings of four flue-cured tobacco varieties gradually decreased with increasing drought stress. Compared with CK, the chlorophyll a content of Q96 did not significantly decrease under D1 treatment, while the chlorophyll a content of Z101, Y87, and Y116 decreased by 7.71%, 14.52%, 18.74%, and 23.27%, respectively. Under D2 treatment, the chlorophyll a content of Q96, Z101, Y87, and Y116 decreased by 18.89%, 26.96%, 31.41%, and 34.33%, respectively. The chlorophyll a content of Q96, Z101, Y87, and Y116 decreased by 31.53%, 36.19%, 38.88%, and 42.56%, respectively, under D3 treatment.

**Table 2:** Effects of different degrees of drought stress on chlorophyll content of flue-cured tobacco seedlings.

Indexes	Treatment	Variety			
		Q96	Z101	Y87	Y116
Chlorophyll a content	CK	1.33 ± 0.06a	1.33 ± 0.09a	1.42 ± 0.04a	1.28 ± 0.04a
	D1	1.23 ± 0.07a	1.13 ± 0.04b	1.15 ± 0.09b	0.98 ± 0.07b
	D2	1.08 ± 0.05b	0.97 ± 0.04c	0.97 ± 0.04c	0.84 ± 0.05c
	D3	0.91 ± 0.03c	0.85 ± 0.02d	0.87 ± 0.03d	0.74 ± 0.04d
Chlorophyll b content	CK	0.28 ± 0.03a	0.27 ± 0.02a	0.24 ± 0.02a	0.25 ± 0.01a
	D1	0.26 ± 0.02a	0.22 ± 0.012b	0.2 ± 0.01b	0.19 ± 0.01b
	D2	0.22 ± 0.02b	0.19 ± 0.01c	0.16 ± 0.01c	0.15 ± 0.02c
	D3	0.19 ± 0.01c	0.15 ± 0.01d	0.14 ± 0.01d	0.11 ± 0.01d
Chlorophyll a+b content	CK	1.62 ± 0.08a	1.60 ± 0.11a	1.66 ± 0.06a	1.53 ± 0.05a
	D1	1.49 ± 0.07a	1.36 ± 0.05b	1.35 ± 0.09b	1.18 ± 0.08b
	D2	1.3 ± 0.05b	1.15 ± 0.05c	1.14 ± 0.04c	0.99 ± 0.05c
	D3	1.10 ± 0.06c	0.10 ± 0.02d	1.00 ± 0.04d	0.85 ± 0.03d

Different lowercase letters in the same column indicate significant differences among different treatments of the same variety (P< 0.05).

Regarding chlorophyll b content, compared with CK treatment, the chlorophyll b content of Q96 did not decrease significantly under D1 treatment, while the chlorophyll b content of Z101, Y87, and Y116 significantly decreased by 8.80%, 18.63%, 19.59%, and 23.19% compared with the control, respectively. Under D2 treatment, the chlorophyll b content of Q96, Z101, Y87, and Y116 decreased by 22.42%, 32.11%, 33.29%, and 40.71%, respectively. The chlorophyll b content of Q96, Z101, Y87, and Y116 decreased by 34.27%, 43.87%, 44.25%, and 54.94%, respectively, under D3 treatment.

For chlorophyll a+b content, compared with CK treatment, the chlorophyll a+b content of Q96 did not significantly decrease under D1 treatment, while the chlorophyll a+b content of Z101, Y87, and Y116 significantly decreased by 7.90%, 15.22%, 18.86%, and 23.26%, respectively compared with the control. Under D2 treatment, the chlorophyll a+b content of Q96, Z101, Y87, and Y116 decreased by 19.51%, 27.84%, 31.68%, and 35.38%, respectively. The chlorophyll a+b content of Q96, Z101, Y87, and Y116 decreased by 32.01%, 37.50%, 39.67%, and 44.60%, respectively, under D3 treatment. The greater the drought stress, the greater the percentage decrease in chlorophyll a, chlorophyll b, and chlorophyll a+b content in the flue-cured tobacco seedlings. According to the degree of chlorophyll decline, the drought resistance of the four flue-cured tobacco varieties ranked as Q96 > Z101 > Y87 > Y116.

### 3.3 Effects of drought stress on photosynthetic parameters of tobacco seedlings

**Figure 1:**
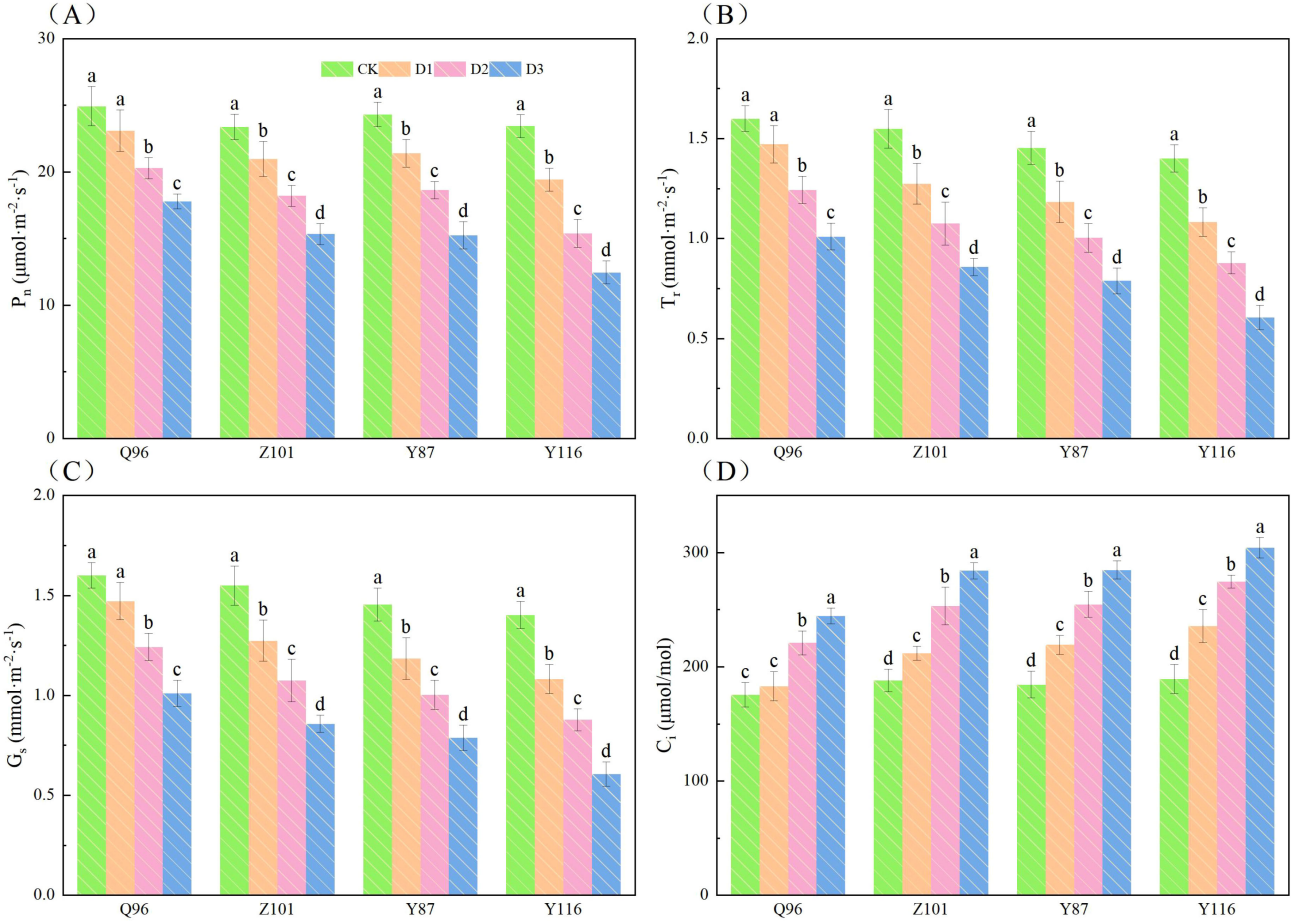
Effects of different drought stress on photosynthetic parameters of tobacco seedlings. **(A)** Effects of different drought stress on P_n_ of tobacco seedlings, **(B)** Effects of different drought stress on T_r_ of tobacco seedlings, **(C)** Effects of different drought stress on G_s_ of tobacco seedlings, and **(D)** Effects of different drought stress on C_i_ of tobacco seedlings. Different lowercase letters in the figure represent significant differences (P <  0.05). “Q96”, “Z101”, “Y87” and “Y116” mean flue-cured tobacco varieties Qinyan 96, Zhongyan 101, Yunyan 87, and Yunyan 116, respectively. CK, D1, D2, D3, represent sufficient water supply (70–75% soil moisture content), mild stress (55–60% soil moisture content), moderate stress (45–50% soil moisture content) and severe stress (35–40% soil moisture content).

**Figure 1** shows that as drought stress increased, the P_n_, T_r_, and G_s_ of the seedlings in all four flue-cured tobacco varieties exhibited a downward trend, while C_i_ showed an upward trend. Compared to CK treatment, the P_n_ (**Figure 1A**) of Q96 did not significantly decrease under D1 treatment, while the P_n_ of Z101, Y87, and Y116 decreased by 7.35%, 10.28%, 11.98%, and 17.11%, respectively. Under D2 treatment, the P_n_ of Q96, Z101, Y87, and Y116 decreased by 18.64%, 22.16%, 23.35%, and 34.41%, respectively. Under D3 treatment, the P_n_ of Q96, Z101, Y87, and Y116 decreased by 28.65%, 34.42%, 37.30%, and 46.84%, respectively.

Compared to CK treatment, the T_r_ (**Figure 1B**) of Q96 did not significantly decrease under D1 treatment, but the T_r_ of Z101, Y87, and Y116 significantly reduced by 8.00%, 17.73%, 18.57%, and 22.73%, respectively. Under D2 treatment, the T_r_ of Q96, Z101, Y87, and Y116 decreased by 22.31%, 30.60%, 31.04%, and 37.29%, respectively. Under D3 treatment, the T_r_ of Q96, Z101, Y87, and Y116 decreased by 36.91%, 44.59%, 45.80%, and 56.71%, respectively.

Compared to CK treatment, the G_s_ (**Figure 1C**) of Q96 did not significantly decrease under D1 treatment, but the G_s_ of Z101, Y87, and Y116 significantly reduced by 8.53%, 12.64%, 16.50%, and 20.26%, respectively. Under D2 treatment, the G_s_ of Q96, Z101, Y87, and Y116 decreased by 18.50%, 27.62%, 31.68%, and 35.87%, respectively. The maximum leaf widths of Q96, Z101, Y87, and Y116 decreased by 33.74%, 39.36%, 44.07%, and 49.81%, respectively, under D3 treatment. As drought stress increased, the greater the percentage decrease in P_n_, T_r_, and G_s_, the worse the drought resistance of the flue-cured tobacco seedlings.

Unlike P_n_, T_r_, and G_s_, the C_i_ (**Figure 1D**) of flue-cured tobacco seedlings increased with the severity of drought stress. The C_i_ of Q96 showed no significant change, while the C_i_ of Z101, Y87, and Y116 increased significantly by 4.22%, 12.66%, 18.91%, and 24.65%, respectively, compared to the control. Under D2 treatment, the C_i_ of Q96, Z101, Y87, and Y116 increased by 25.94%, 34.67%, 38.17%, and 45.18%, respectively. Under D3 treatment, the C_i_ of Q96, Z101, Y87, and Y116 increased by 39.30%, 51.03%, 54.49%, and 60.87%, respectively. As drought stress increased, the greater the percentage increase in C_i_ of flue-cured tobacco seedlings, the lower their drought resistance. Based on the decrease in P_n_, T_r_, and G_s_, and the increase in C_i_, the drought resistance of the four flue-cured tobacco varieties ranked as follows: Q96 > Z101 > Y87 > Y116.

### 3.4 Effects of drought stress on chlorophyll fluorescence parameters in tobacco seedlings

**Figure 2:**
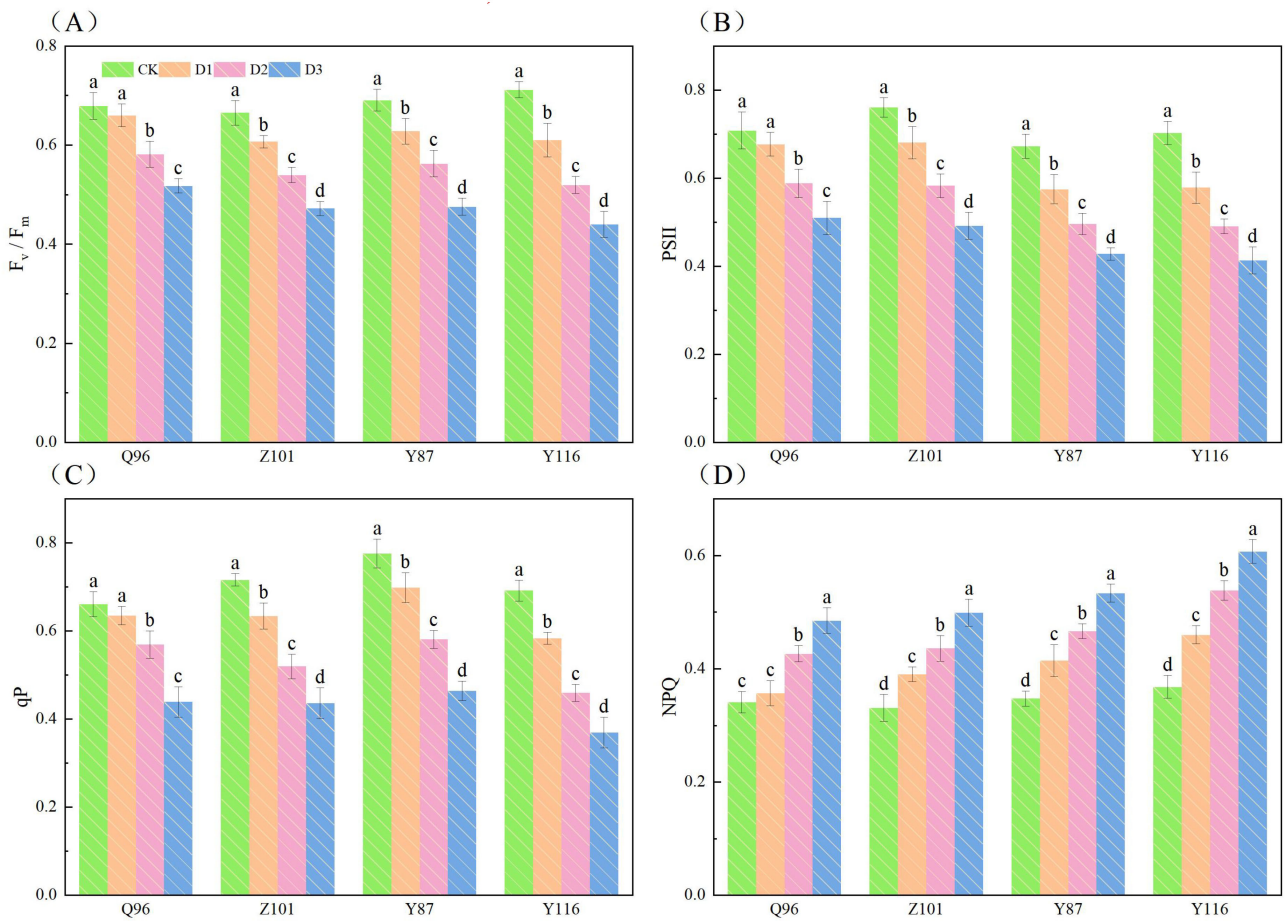
Effects of different drought stress on chlorophyll fluorescence system of tobacco seedlings. **(A)** Effects of different drought stress on F_v_/F_m_ of tobacco seedlings, **(B)** Effects of different drought stress on PSII of tobacco seedlings, **(C)** Effects of different drought stress on qP of tobacco seedlings, and **(D)** Effects of different drought stress on NPQ of tobacco seedlings. Different lowercase letters in the figure represent significant differences (P <  0.05). “Q96”, “Z101”, “Y87” and “Y116” mean flue-cured tobacco varieties Qinyan 96, Zhongyan 101, Yunyan 87, and Yunyan 116, respectively. CK, D1, D2, D3, represent sufficient water supply (70–75% soil moisture content), mild stress (55–60% soil moisture content), moderate stress (45–50% soil moisture content) and severe stress (35–40% soil moisture content).

**Figure 2** shows that with increased drought stress, F_v_/F_m_, PSII, and qP of the seedlings of the four flue-cured tobacco varieties gradually decreased, while NPQ gradually increased. In terms of F_v_/F_m_ (**Figure 2A**), compared with CK treatment, the Fv/Fm of Q96 showed no significant decrease under D1 treatment, while the Fv/Fm of Z101, Y87, and Y116 decreased significantly by 2.80%, 8.68%, 9.07%, and 14.24%, respectively, compared to their respective controls. Under D2 treatment, the F_v_/F_m_ of Q96, Z101, Y87, and Y116 decreased by 14.29%, 18.81%, 18.53%, and 27.03%, respectively. Under D3 treatment, the F_v_/F_m_ of Q96, Z101, Y87, and Y116 decreased by 23.72%, 28.99%, 31.13%, and 38.22%, respectively.

Compared with CK treatment, the PSII (**Figure 2B**) of Q96 did not significantly decrease under D1 treatment, while the PSII of Z101, Y87, and Y116 significantly decreased by 4.38%, 10.44%, 14.52%, and 17.66%, respectively, compared to their controls. Under D2 treatment, the PSII of Q96, Z101, Y87, and Y116 decreased by 16.95%, 23.33%, 26.26%, and 30.19%, respectively. The PSII of Q96, Z101, Y87, and Y116 further decreased by 28.01%, 35.31%, 36.37%, and 41.20%, respectively, under D3 treatment.

Compared with CK treatment, the qP (**Figure 2C**) of Q96, Z101, Y87, and Y116 decreased by 3.98%, 11.36%, 10.01%, and 15.61% under D1 treatment. Under D2 treatment, the qP of Q96, Z101, Y87, and Y116 decreased by 13.87%, 27.39%, 25.13%, and 33.54%, respectively. The qP of Q96, Z101, Y87, and Y116 further decreased by 33.59%, 39.08%, 40.21%, and 46.55%, respectively, under D3 treatment. As drought stress intensified, the greater the percentage decrease in F_v_/F_m_, PSII, and qP of flue-cured tobacco seedlings, the worse their drought resistance.

Compared with CK treatment, the NPQ (**Figure 2D**) of Q96 did not significantly increase under D1 treatment, but the NPQ of Z101, Y87, and Y116 significantly increased by 4.49%, 18.04%, 19.29%, and 25.00%, respectively, compared to the control. Under D2 treatment, the NPQ of Q96, Z101, Y87, and Y116 increased by 25.00%, 31.85%, 34.26%, and 46.29%, respectively. The NPQ of Q96, Z101, Y87, and Y116 further increased by 42.09%, 50.91%, 53.65%, and 65.04%, respectively, under D3 treatment. As drought stress intensified, the greater the percentage increase in NPQ of flue-cured tobacco seedlings, the worse their drought resistance. Based on the decreasing degree of F_v_/F_m_, PSII, and qP, and the increase of NPQ, the drought resistance of the four flue-cured tobacco varieties was ranked as Q96 > Z101 > Y87 > Y116.

### 3.5 The effect of drought stress on the superoxide anion free radical production rate in tobacco seedlings

**Figure 3:**
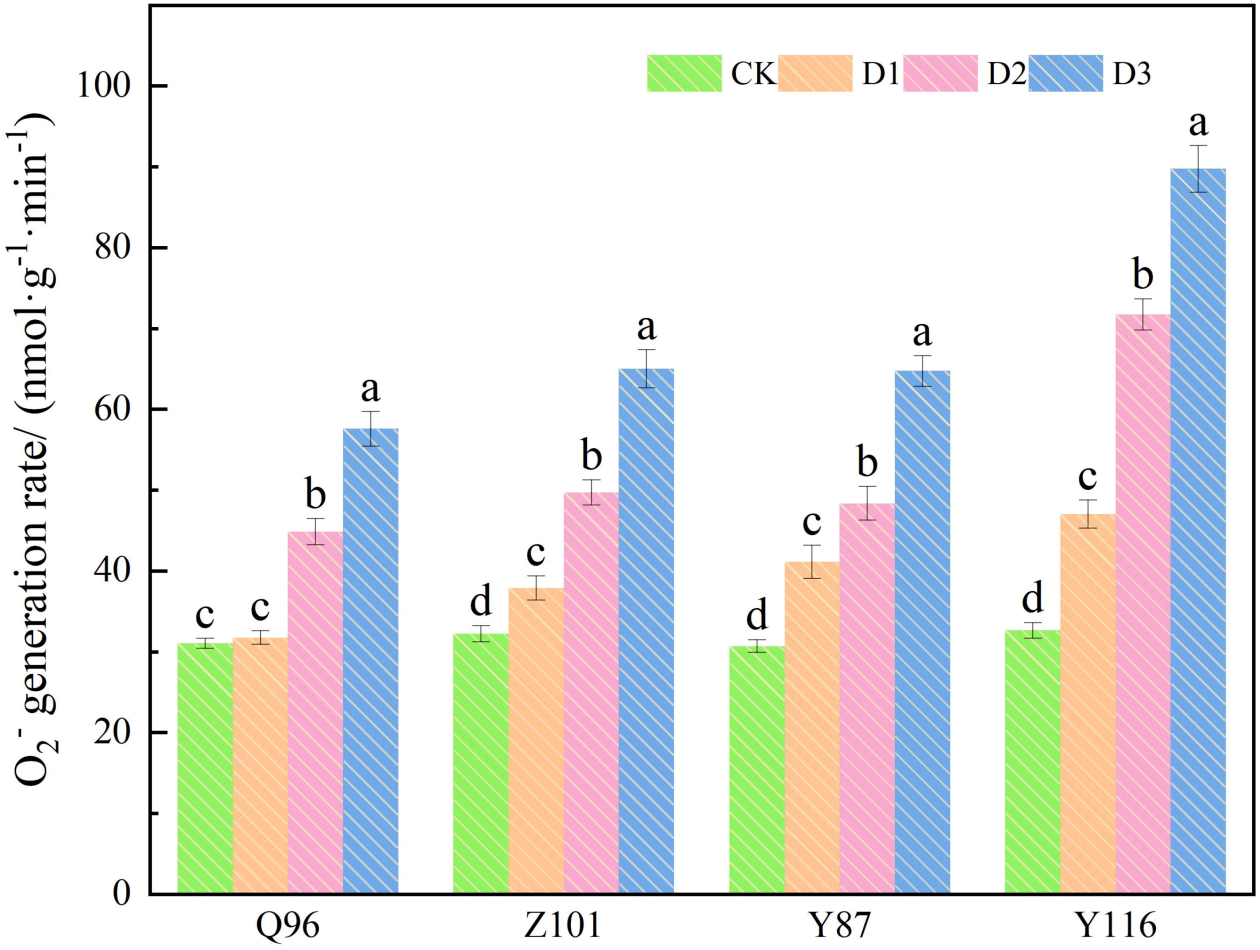
Effects of different drought stress on O_2_^-^ generation rate of tobacco seedlings. Different lowercase letters in the figure represent significant differences (P <  0.05). “Q96”, “Z101”, “Y87” and “Y116” mean flue-cured tobacco varieties Qinyan 96, Zhongyan 101, Yunyan 87, and Yunyan 116, respectively. CK, D1, D2, D3, represent sufficient water supply (70–75% soil moisture content), mild stress (55–60% soil moisture content), moderate stress (45–50% soil moisture content) and severe stress (35–40% soil moisture content).

As shown in **Figure 3**, the O_2_^-^ production rate of the four flue-cured tobacco varieties increased with the severity of drought stress. Compared with the CK treatment, the O_2_^-^ production rate of Q96 did not increase significantly under D1 treatment, while the O_2_^-^ production rates of Z101, Y87, and Y116 increased significantly, by 2.31%, 17.55%, 34.09%, and 44.05%, respectively. Under D2 treatment, the O_2_^-^ production rates of Q96, Z101, Y87, and Y116 increased by 44.43%, 54.23%, 57.55%, and 119.66%, respectively. The O_2_^-^ production rates of Q96, Z101, Y87, and Y116 increased by 85.45%, 101.65%, 110.93%, and 174.90% under D3 treatment, respectively. As drought stress intensified, the greater the percentage increase in O_2_^-^ production rate in flue-cured tobacco seedlings, the lower their drought resistance. The drought resistance of the four flue-cured tobacco varieties was ranked as Q96 > Z101 > Y87 > Y116 based on the increase in O_2_^-^ production rate.

### 3.6 Effects of drought stress on MDA content in flue-cured tobacco seedlings

**Figure 4:**
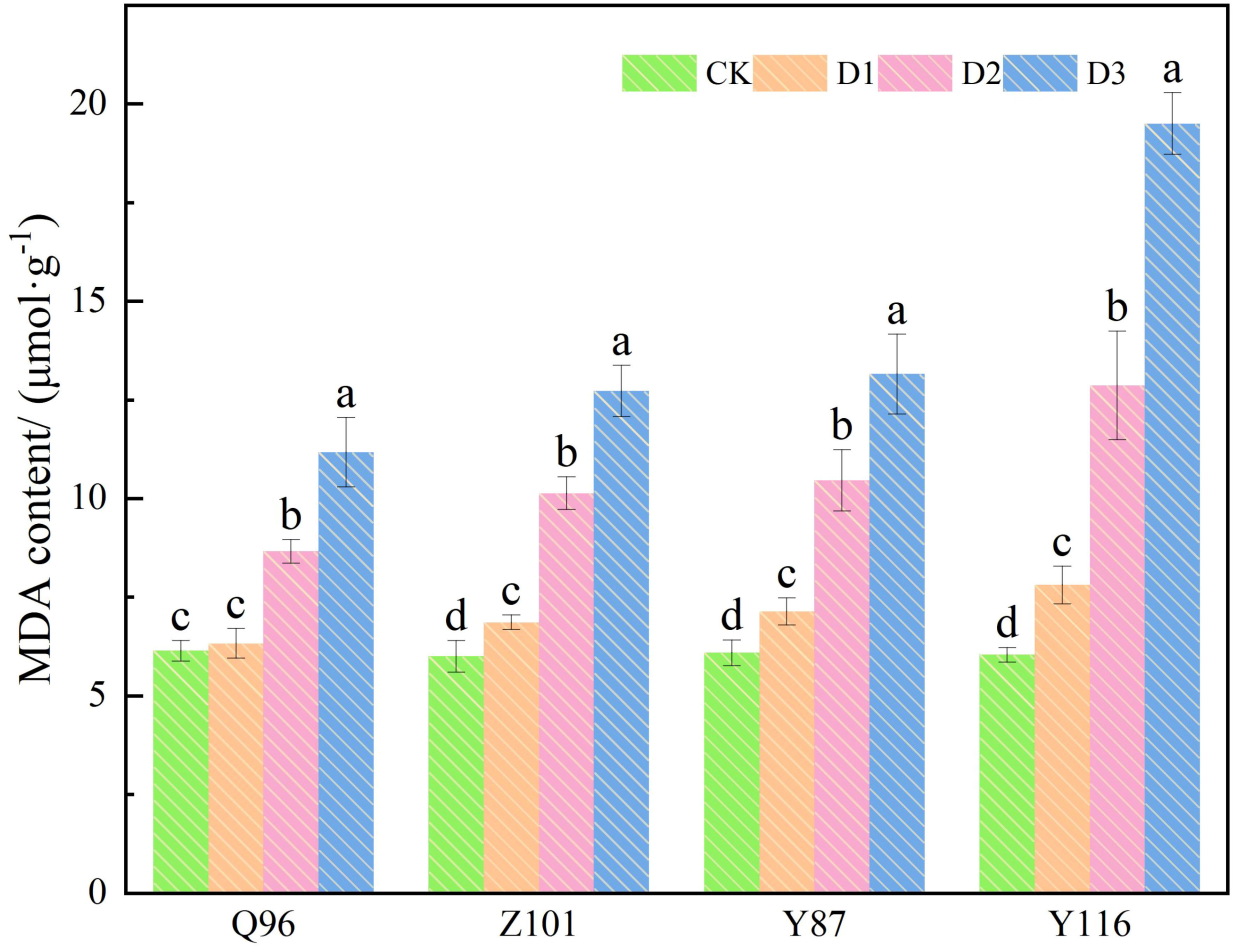
Effects of different drought stress on MDA content of tobacco seedlings. Different lowercase letters in the figure represent significant differences (P <  0.05). “Q96”, “Z101”, “Y87” and “Y116” mean flue-cured tobacco varieties Qinyan 96, Zhongyan 101, Yunyan 87, and Yunyan 116, respectively. CK, D1, D2, D3, represent sufficient water supply (70–75% soil moisture content), mild stress (55–60% soil moisture content), moderate stress (45–50% soil moisture content) and severe stress (35–40% soil moisture content).

As shown in **Figure 4**, the MDA content of the four flue-cured tobacco varieties also increased with drought stress. Compared with the CK treatment, the MDA content of Q96 did not increase significantly under D1 treatment, while the MDA contents of Z101, Y87, and Y116 increased significantly, by 3.09%, 14.40%, 17.23%, and 29.26%, respectively, compared to the control. Under D2 treatment, the MDA contents of Q96, Z101, Y87, and Y116 increased by 41.07%, 68.79%, 71.61%, and 112.92%, respectively. The MDA contents of Q96, Z101, Y87, and Y116 increased by 81.93%, 111.94%, 115.95%, and 222.78%, respectively, under D3 treatment. As drought stress intensified, the greater the percentage increase in MDA content in flue-cured tobacco seedlings, the lower their drought resistance. The drought resistance of the four flue-cured tobacco varieties was ranked as Q96 > Z101 > Y87 > Y116 based on the increase in MDA content.

### 3.7 Effects of drought stress on antioxidant enzyme activity in tobacco seedlings

**Figure 5:**
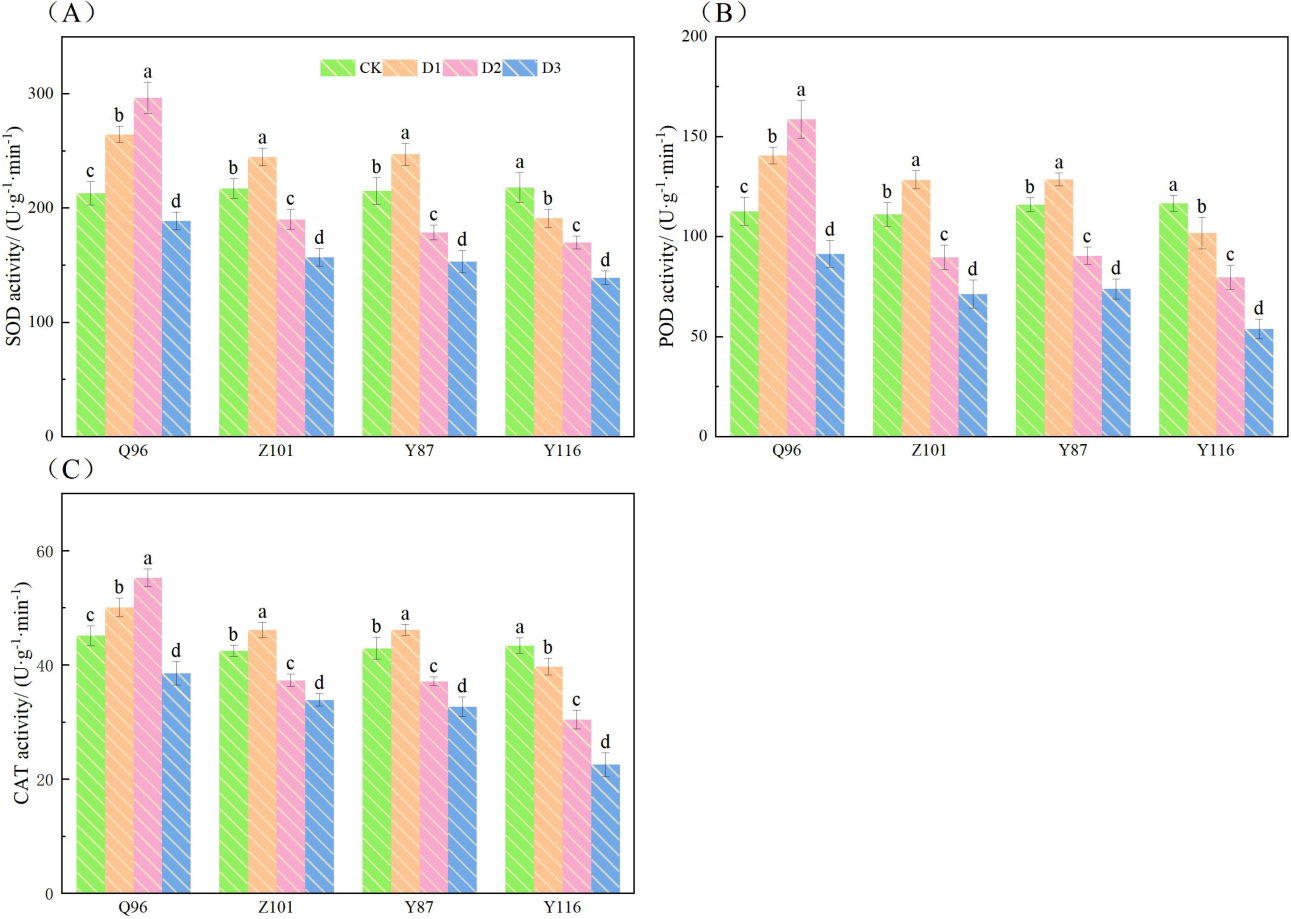
Effects of different drought stress on the activities of SOD, POD, and CAT of tobacco seedlings **(A)** Effects of different drought stress on SOD activities of tobacco seedlings, **(B)** Effects of different drought stress on POD activities of tobacco seedlings, and **(C)** Effects of different drought stress on CAT activities of tobacco seedling. Different lowercase letters in the figure represent significant differences (P <  0.05). “Q96”, “Z101”, “Y87” and “Y116” mean flue-cured tobacco varieties Qinyan 96, Zhongyan 101, Yunyan 87, and Yunyan 116, respectively. CK, D1, D2, D3, represent sufficient water supply (70–75% soil moisture content), mild stress (55–60% soil moisture content), moderate stress (45–50% soil moisture content) and severe stress (35–40% soil moisture content).

As shown in **Figure 5**, compared with the CK treatment, the SOD (**Figure 5A**) of Q96, Z101, and Y87 tobacco seedlings under D1 treatment increased by 24.19%, 12.79%, and 14.95%, respectively, while the SOD of Y116 decreased by 12.33%. Under D2 treatment, the SOD of Z101, Y87, and Y116 increased by 39.19%, 12.44%, 16.91%, and 22.09%, respectively. However, under D3 treatment, the SOD of Q96, Z101, Y87, and Y116 decreased by 11.43%, 27.68%, 28.76%, and 36.23%, respectively.

Compared with the CK treatment, the POD (**Figure 5B**) of Q96, Z101, and Y87 tobacco seedlings increased by 24.91%, 15.63%, and 10.96%, respectively, while the POD of Y116 decreased by 12.83%. Under D2 treatment, the POD increased by 40.96% for all but Q96, which saw a decrease of 19.31%, 21.97%, and 31.79% for Z101, Y87, and Y116, respectively. Under D3 treatment, the POD of Q96, Z101, Y87, and Y116 decreased by 18.77%, 35.86%, 36.37%, and 53.82%, respectively.

Compared with the CK treatment, the CAT (**Figure 5C**) of Q96, Z101, and Y87 tobacco seedlings increased by 10.88%, 8.43%, and 7.49%, respectively, while the CAT of Y116 decreased by 8.41%. Under D2 treatment, the CAT of Q96 increased by 22.48%, while Z101, Y87, and Y116 decreased by 12.20%, 13.48%, and 29.87%, respectively. However, under D3 treatment, the CAT of Q96, Z101, Y87, and Y116 decreased by 14.65%, 20.27%, 23.89%, and 48.08%, respectively.

Under D1 treatment, the values of SOD, POD, and CAT decreased only in Y116 compared with CK, indicating that Y116 exhibited poor drought resistance under mild drought stress. Under D2 treatment, SOD, POD, and CAT increased only in Q96 compared with CK, indicating that Q96 showed strong drought resistance under moderate drought stress. Under D3 treatment, the SOD, POD, and CAT values of all four flue-cured tobacco varieties decreased, reflecting a reduction in the antioxidant enzyme system when drought stress reached a certain level. In conclusion, the results showed that as drought stress intensified, the SOD, POD, and CAT levels in the seedlings of the four flue-cured tobacco varieties gradually declined. The greater the decline, the worse the drought resistance of the varieties, in the following order: Q96 > Z101 > Y87 > Y116.

### 3.8 Comprehensive evaluation of drought resistance in seedlings of different tobacco varieties

**Table 3** shows that 21 individual indicators were determined by principal component analysis (PCA). The number of principal components was determined based on an eigenvalue ≥ 1.00, with principal components having a cumulative contribution rate of ≥ 85% used as the comprehensive index to evaluate the drought resistance of flue-cured tobacco. Under CK treatment, the variance contribution rates of the first six principal components were 26.52%, 19.61%, 15.75%, 12.19%, 7.17%, and 6.38%, respectively, with a cumulative contribution rate of 87.62%. This transformed 21 individual indicators into six comprehensive indicators, with the first principal component contributing over 25%. Under D1 treatment, the variance contribution rates of the first five principal components were 61.37%, 11.21%, 7.22%, 5.29%, and 4.97%, respectively, with a cumulative contribution rate of 90.06%, resulting in five comprehensive indicators. Under D2 treatment, the variance contribution rates of the first four principal components were 66.60%, 10.22%, 6.67%, and 4.84%, respectively, with a cumulative contribution rate of 88.33%, resulting in four comprehensive indicators. Under D3 treatment, the variance contribution rates of the first four principal components were 72.59%, 8.49%, 5.64%, and 4.90%, respectively, with a cumulative contribution rate of 88.33%, also resulting in four comprehensive indicators. Unlike the CK treatment, the first principal component in the D1, D2, and D3 treatments all exceeded 60%. The PCA results reflect the function of the drought resistance index of flue-cured tobacco varieties and can comprehensively evaluate the differences in drought resistance among these varieties.

**Table 3:** Principal component analysis of drought resistance in tobacco seedlings under drought stress.

Treatment	Principal component	Indexes			
		Eigenvalue	CR (%)	CCR (%)	W_j_
CK	1	5.57	26.52	26.52	0.30
	2	4.12	19.61	46.13	0.22
	3	3.31	15.75	61.88	0.18
	4	2.56	12.19	74.07	0.14
	5	1.51	7.17	81.23	0.08
	6	1.34	6.38	87.62	0.07
D1	1	12.89	61.37	61.37	0.68
	2	2.35	11.21	72.58	0.12
	3	1.52	7.22	79.80	0.08
	4	1.11	5.29	85.09	0.06
	5	1.04	4.97	90.06	0.06
D2	1	13.99	66.60	66.60	0.75
	2	2.15	10.22	76.82	0.12
	3	1.40	6.67	83.49	0.08
	4	1.02	4.84	88.33	0.05
D3	1	15.24	72.59	72.59	0.79
	2	1.78	8.49	81.07	0.09
	3	1.19	5.64	86.72	0.06
	4	1.03	4.90	91.62	0.05

CR, contribution rate; CCR and cumulative contribution rate.

### 3.9 Screening of drought-resistant traits in tobacco seedlings under drought stress

**Figure 6:**
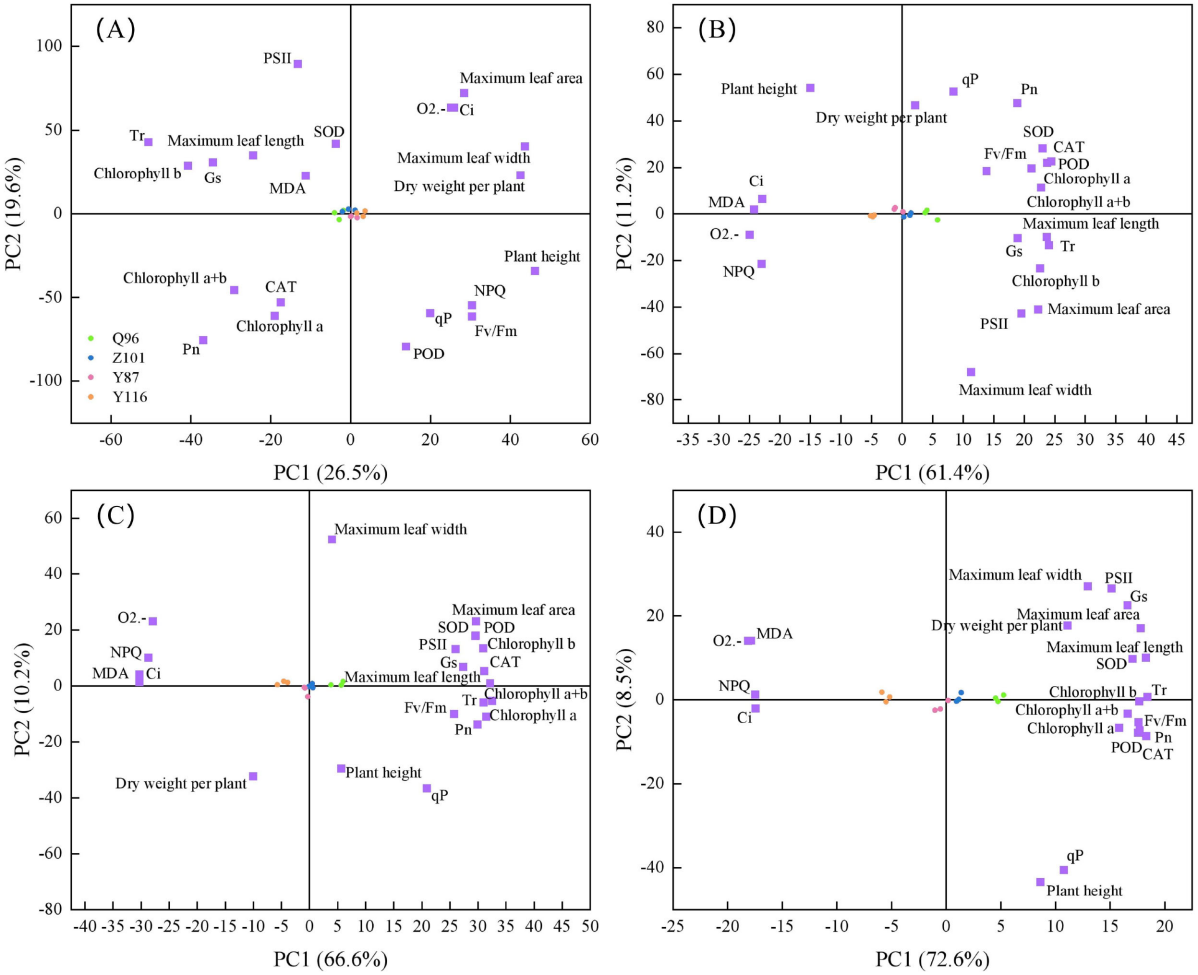
Screening of 21 drought resistant traits in different varieties of tobacco seedlings under different drought stresses. **(A)** Screening of 21 drought resistant traits in different varieties of tobacco seedlings under CK treatment, **(B)** Screening of 21 drought resistant traits in different varieties of tobacco seedlings under D1 treatment, **(C)** Screening of 21 drought resistant traits in different varieties of tobacco seedlings under D2 treatment, and **(D)** Screening of 21 drought resistant traits in different varieties of tobacco seedlings under D3 treatment.

Here, 21 drought resistance traits were evaluated under CK, D1, D2, and D3 treatments, with the distribution of each trait shown in **Figure 6**. There was a positive correlation between parallel indicators in the PCA plot, i.e., close traits, and a negative correlation between opposite traits. Under the CK (**Figure 6A**) treatment, the contribution rates of PC1 and PC2 were 26.50% and 19.60%, respectively, explaining 46.10% of the total variation. PC1 was dominated by plant height, maximum leaf width, dry weight per plant, and Tr, while PC2 was dominated by PSII, POD, and P_n_. Under the D1 (**Figure 6B**) treatment, the contribution rates of PC1 and PC2 were 61.40% and 11.20%, respectively, explaining 72.60% of the total variation. PC1 was dominated by POD, T_r_, MDA, and O2.-, while PC2 was dominated by qP, plant height, maximum leaf width, and P_n_. Under the D2 treatment (**Figure 6C**), the contribution rates of PC1 and PC2 were 66.60% and 10.20%, respectively, accounting for 76.80% of the total variation. PC1 was dominated by chlorophyll a+b, maximum leaf length, MDA, and NPQ, while PC2 was dominated by maximum leaf width and qP. Under the D3 (**Figure 6D**) treatment, the contribution rates of PC1 and PC2 were 72.60% and 8.50%, respectively, explaining a total of 81.10% of the variation. PC1 was dominated by T_r_, P_n_, and maximum leaf area, while PC2 was dominated by maximum leaf width, PSII, POD, qP, and plant height.

### 3.10 Comprehensive evaluation of drought tolerance of tobacco seedlings under drought stress

**Figure 7:**
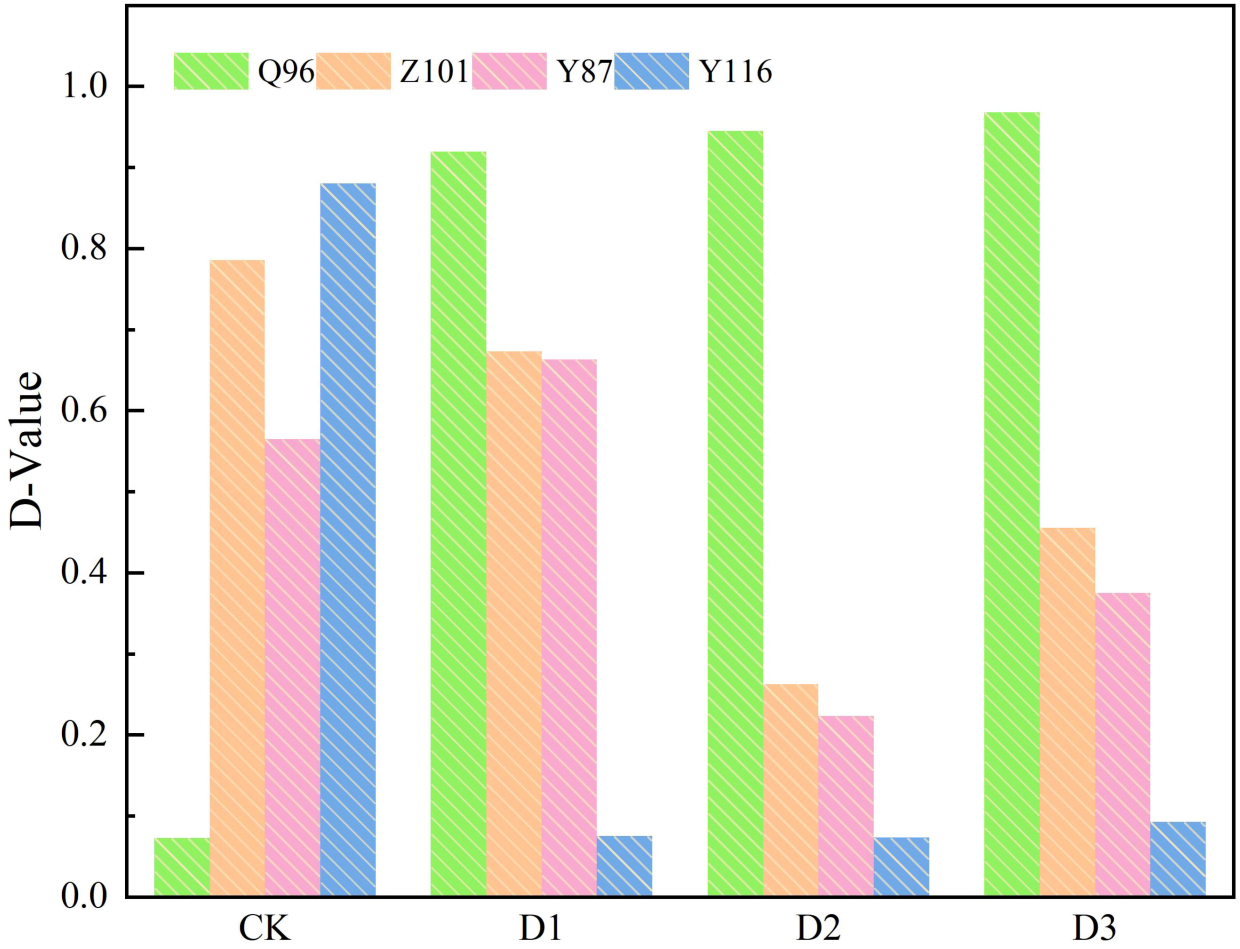
D values of tobacco seedlings of four varieties under different drought stress. “Q96”, “Z101”, “Y87” and “Y116” mean flue-cured tobacco varieties Qinyan 96, Zhongyan 101, Yunyan 87, and Yunyan 116, respectively. CK, D1, D2, D3, represent sufficient water supply (70–75% soil moisture content), mild stress (55–60% soil moisture content), moderate stress (45–50% soil moisture content) and severe stress (35–40% soil moisture content).

According to equation (5), the membership function of each composite index for the four flue-cured tobacco varieties (**Table 4**) was determined, and the weight of each composite index (**Table 3**) was calculated using equation (6). The weights of the six composite indicators were 0.30, 0.22, 0.18, 0.14, 0.08, and 0.07 under the CK treatment; 0.68, 0.12, 0.08, 0.06, and 0.06 for the five composite indicators under the D1 treatment; 0.75, 0.12, 0.08, and 0.05 for the four composite indicators under the D2 treatment; and 0.79, 0.09, 0.06, and 0.05 for the four composite indicators under the D3 treatment. Finally, the D value for the comprehensive evaluation of drought resistance of each tobacco variety was calculated using formula (7). This D value, which integrates various drought resistance traits, is shown in **Figure 7**. The drought resistance of the flue-cured tobacco varieties was compared according to the D value. The D value of Q96 was smaller under CK treatment, indicating weaker drought resistance. However, the D value tended to be 1.00 under D1, D2, and D3 treatments, demonstrating stronger drought resistance compared to the other three varieties. Under the three drought stresses of D1, D2, and D3, Z101 exhibited weaker drought resistance than Q96, Y87 showed weaker drought resistance than Z101, and Y116 had the weakest drought resistance compared to the other three flue-cured tobacco varieties. Therefore, the drought resistance of the four flue-cured tobacco varieties under D1, D2, and D3 was ranked as follows: Q96 > Z101 > Y87 > Y116.

**Table 4:** The membership function values of comprehensive indicators of different tobacco seedlings under drought stress.

Treatment	Membership function value	Variety			
		Q96	Z101	Y87	Y116
CK	U(X_1_)	0.00	0.75	0.62	1.00
	U(X_2_)	0.00	1.00	0.36	0.77
	U(X_3_)	0.00	1.00	0.69	0.99
	U(X_4_)	0.00	0.61	0.88	1.00
	U(X_5_)	0.00	0.38	0.59	1.00
	U(X_6_)	1.00	0.53	0.00	0.07
D1	U(X_1_)	1.00	0.63	0.56	0.00
	U(X_2_)	1.00	0.75	0.88	0.00
	U(X_3_)	0.00	0.84	1.00	0.94
	U(X_4_)	1.00	0.76	0.79	0.00
	U(X_5_)	1.00	0.75	0.78	0.00
D2	U(X_1_)	1.00	0.28	0.23	0.00
	U(X_2_)	1.00	0.09	0.00	0.08
	U(X_3_)	1.00	0.00	0.00	0.12
	U(X_4_)	0.00	0.82	0.86	1.00
D3	U(X_1_)	1.00	0.50	0.46	0.00
	U(X_2_)	0.66	0.38	0.00	1.00
	U(X_3_)	1.00	0.22	0.21	0.00
	U(X_4_)	1.00	0.15	0.03	0.00

## 4 Discussion

Drought stress significantly impedes plant growth and development, primarily due to photosynthesis limitations, disruptions in internal metabolic systems such as antioxidant systems, and other physiological performance degradation induced by drought stress (Ilyas et al., 2021). This study demonstrates that escalating drought severity inhibits seedling growth and development across all four tobacco varieties, albeit to varying degrees. Q96 exhibits a relatively minor growth decrease, whereas Y116 displays the most significant reduction, indicating that different drought-tolerant varieties respond distinctly to drought stress. Drought-tolerant plants rapidly adapt to arid conditions and enhance water use efficiency through self-regulation, a hallmark of drought resistance (Wahab et al., 2022). Accordingly, drought-resistant tobacco varieties exhibit smaller changes in growth indicators under drought stress than water-sensitive varieties, reflecting their genotypic advantages.

Through photosynthesis, plants accumulate essential substances and energy for growth and development (Zhen et al., 2022). Chlorophyll, predominantly found in leaves, facilitates light energy absorption for photosynthesis, with its concentration influencing the photosynthetic energy levels. Therefore, chlorophyll significantly impacts the internal light energy transfer within plants (Porcar-Castell et al., 2021; Simkin et al., 2022; Wang et al., 2022a). Given its sensitivity to drought stress, chlorophyll synthesis is considerably inhibited under such conditions. Severe drought stress can even trigger chlorophyll decomposition in plants (Khan et al., 2023). This experiment’s findings align with previous studies, confirming that escalating drought severity correlates with decreased chlorophyll content in tobacco seedling leaves (Jiang-Bo et al., 2013).

Upon experiencing drought stress, crops exhibit a decrease in water potential, which safeguards stomatal cells, resulting in stomatal closure within the leaves and impeding CO_2_ absorption and utilization. During this phase, weakened photosynthesis serves as the stomatal limitation. Prolonged drought stress causes the closure of some or all stomata, elevating CO_2_ concentration in leaf tissue, and damaging chloroplast structure, causing a decrease in chlorophyll content (Nagamalla et al., 2021). This damage leads to thylakoid membrane disintegration and the impairment of PSII function, consequently reducing the photosynthetic performance of mesophyll cells. At this stage, the non-stomatal factors, specifically the diminished photosynthetic capacity of plant mesophyll cells, cause the photosynthesis to decline (Sharma et al., 2020). This experiment’s findings demonstrated that drought stress decreased P_n_, T_r_, and G_s_ in two tobacco varieties, aligning with previous research (Wen-Quan et al., 2013) on the small crown flower, while C_i_ increased. The rehydration of drum bamboo following drought stress suggests that the reduced photosynthetic rate in tobacco is attributable to non-stomatal factors. Drought-resistant tobacco varieties rapidly acclimate to drought stress via self-regulation, mitigating drought’s adverse effects on the tobacco’s photosynthetic system. Severe drought stress significantly impacts the chlorophyll fluorescence parameters of tobacco leaves, causing decreases in F_v_/F_m_ and qP, thereby limiting normal photosynthesis progression. Upon photosynthetic system damage, tobacco initiates self-protection mechanisms, which safeguard the photosynthetic system by promptly increasing NPQ and dissipating excess light energy (Begum et al., 2020; Chen et al., 2020). This study’s findings demonstrate that escalating drought stress levels correspond with reduced F_v_/F_m_, PSII, and qP, all significantly below control levels, while NPQ increases surpassing control levels.

Drought stress induces the formation of substantial reactive oxygen species (ROS) in plant cells. Delayed cleanup can trigger oxidative damage, impairing the membrane system and instigating membrane lipid peroxidation. O_2_^-^, a reactive oxygen species derivative formed by O_2_^-^ gaining an electron, inflicts damage on plant tissues and cell membranes (Guo et al., 2019). Upon exposure to external drought stress, protective enzymes within plants synergistically suppress the extensive production of the lipid peroxidation product, malondialdehyde (MDA). As a primary peroxidation product, MDA undermines the structural functions of nucleic acids, proteins, and cell membranes, significantly affecting plant growth (Sarker and Oba, 2018). This experiment’s findings suggest that escalating drought stress correlates with a significant increase in ROS accumulation in tobacco seedlings. Both the O_2_^-^ production rate and MDA content of Q96 and Z101 were significantly lower than those of Y87 and Y116, indicating a superior antioxidant capacity in Q96 and Z101.

Under drought stress, plants can enhance antioxidant enzyme activity to achieve self-protection and reduce damage (Dvorák et al., 2021; Rajput et al., 2021; Mishra et al., 2023). If the drought severity does not exceed the plant cells’ tolerance range, the enhanced antioxidant enzyme activity can effectively reduce free radical damage. However, exceeding this range disrupts the reactive oxygen species balance system, weakening antioxidant enzyme activity and causing ROS accumulation to exceed clearance capacity, resulting in plant cell damage. In this experiment, mild drought stress increased antioxidant enzyme activity in Q96, Z101, and Y87, but decreased it in Y116. This indicates that mild drought stress did not exceed the tolerance range of the first three tobacco varieties, allowing them to regulate the impact of drought stress on the reactive oxygen species system by enhancing their antioxidant enzyme activity. The antioxidant enzyme activity of Q96 peaked under moderate drought stress, while that of Z101 and Y87 peaked under mild drought stress. This suggests that although Q96 has a broad drought stress tolerance, the antioxidant systems of Z101 and Y87 have been damaged under moderate drought stress, surpassing their resistance thresholds. Therefore, they cannot efficiently eliminate ROS. Furthermore, the ROS system of Y116 is already impaired under mild drought stress, indicating lower drought stress tolerance. These findings align with the research of Selwal et al. (Selwal et al., 2022). on various tartary buckwheat varieties.

Plant drought resistance is a complex trait affected by genetic characteristics and external environment. Evaluating drought resistance through a single index is problematic, as it is only reflected by various indicators such as growth and development, photosynthetic characteristics, fluorescence characteristics, reactive oxygen species metabolism, and antioxidant enzyme activity. Each index’s response to drought stress is inconsistent and often cannot accurately reflect drought resistance. Jang et al. (2024) suggested that the fresh dry weight of leaves and stems, relative water content, and SPAD value were key components of the visual score for Chinese cabbage wilting. PCA can convert multiple single indicators into a few comprehensive indicators, effectively avoiding missing data and classifying drought-resistant plant genotypes (Wu and Bao, 2012). Li et al. (2023) used PCA and membership function analysis to evaluate the drought tolerance of lettuce cultivars, selecting surface area (RSA), root volume (RV), underground dry weight (BDW), and soluble sugar (SS) for assessing lettuce genotype drought resistance. The cumulative contribution rate of comprehensive indicators under the CK, D1, D2, and D34 water gradients exceeded 85%, covering most of the data represented by the 21 single indicators. The contribution of the indicators to drought resistance in flue-cured tobacco varied under different drought conditions. The membership function values of four flue-cured tobacco varieties were calculated using the principal component scores, and the D values for the comprehensive evaluation of drought resistance were determined by combining these scores with the weights to perform the evaluation. The results showed that, among the three drought stress treatments, Q96 exhibited strong drought resistance, followed by Z101 and Y87, while Y116 displayed weak drought resistance. However, this study only analyzed and evaluated the drought resistance of flue-cured tobacco seedlings through simulated drought stress experiments. The actual drought resistance of flue-cured tobacco varieties in the field and throughout the entire growth period still needs further verification. Additionally, since drought tolerance traits in plants are typically controlled by multiple genes and the drought resistance mechanism is complex, further research is needed to explore the drought resistance mechanisms of different flue-cured tobacco varieties at the molecular and genetic levels.

## 5 Conclusion

The results showed that varying degrees of drought stress inhibited the growth and development of tobacco and significantly affected the physiological and metabolic activities of flue-cured tobacco seedlings. However, the responses of different varieties varied in agronomic traits, photosynthesis, chlorophyll fluorescence characteristics, reactive oxygen species metabolism, and antioxidant enzyme activity. Principal component analysis (PCA) and membership function analysis were combined to evaluate the drought resistance of flue-cured tobacco. Under D1 treatment, peroxidase (POD), T_r_, malondialdehyde (MDA), and O_2_^.-^ significantly contributed to the drought resistance of flue-cured tobacco (61.40%). Under D2 treatment, Chlorophyll a+b, maximum leaf length, MDA, and NPQ significantly contributed to drought resistance (66.60%), while under D3 treatment, Tr, P_n_, and maximum leaf area were significant contributors (72.60%). Based on various physiological indices of four tobacco cultivars, drought resistance under different drought stress levels was ranked as follows: Q96 > Z101 > Y87 > Y116. Using comprehensive indicators provides important support for accurately evaluating flue-cured tobacco’s response to drought stress, selecting drought-resistant varieties, and offering a theoretical basis for exploring the molecular mechanisms of drought resistance in flue-cured tobacco.

## Data Availability

The raw data supporting the conclusions of this article will be made available by the authors, without undue reservation.
